# Retraction Note: Optimizing microbial strain selection for pyrethroid biodegradation in contaminated environments through a TOPSIS-based decision-making system

**DOI:** 10.1038/s41598-026-36753-2

**Published:** 2026-01-21

**Authors:** Saurabh Gangola, Shshank Chaube, Abdullah Bayram, Samiksha Joshi, Geeta Bhandari, Sumira Malik, Azmat Ali Khan

**Affiliations:** 1 School of Agriculture, Graphic Era Hill University, Bhimtal, 263132 India; 2 Department of Mathematics, Jaypee University, Anoopshar, 203390 Uttar Pradesh India; 3 Department of Agricultural & Biological Engineering, Purdue University, West Lafayette, IN 47906 USA; 4 Department of Biosciences, Swami Rama Himalayan University, Dehradun, 248140 India; 5 Amity Institute of Biotechnology, Amity University Jharkhand, Ranchi, 834001 Jharkhand India; 6 School of Applied and Life Sciences, Uttaranchal University, Dehradun, 248007 Uttarakhand India; 7 Pharmaceutical Biotechnology Laboratory, Department of Pharmaceutical Chemistry, College of Pharmacy, King Saud University, 11451 Riyadh, Saudi Arabia

Retraction of: *Scientific Reports* 10.1038/s41598-024-59223-z, published online 28 June 2024

Editors have retracted this Article as concerns have been raised regarding the authorship of this Article. As the provenance of this research cannot be confirmed, Editors have lost confidence in the reliability of this Article.

Saurabh Gangola disagrees with this retraction. Shshank Chaube, Abdullah Bayram, Samiksha Joshi, Geeta Bhandari, Sumira Malik, and Azmat Ali Khan did not respond to the Editors’ correspondence about this retraction.

